# Predicting water-to-cyclohexane partitioning of the SAMPL5 molecules using dielectric balancing of force fields

**DOI:** 10.1007/s10822-016-9950-z

**Published:** 2016-08-29

**Authors:** S. Shanaka Paranahewage, Cassidy S. Gierhart, Christopher J. Fennell

**Affiliations:** 1 Department of Chemistry, Oklahoma State University, Stillwater, OK 74078 USA; 2 Department of Chemical Engineering, Oklahoma State University, Stillwater, OK 74078 USA

**Keywords:** SAMPL, Force field, Solvation free energy, Dielectric constant, Distribution coefficient

## Abstract

Alchemical transformation of solutes using classical fixed-charge force fields is a popular strategy for assessing the free energy of transfer in different environments. Accurate estimations of transfer between phases with significantly different polarities can be difficult because of the static nature of the force fields. Here, we report on an application of such calculations in the SAMPL5 experiment that also involves an effort in balancing solute and solvent interactions via their expected static dielectric constants. This strategy performs well with respect to predictive accuracy and correlation with unknown experimental values. We follow this by performing a series of retrospective investigations which highlight the potential importance of proper balancing in these systems, and we use a null hypothesis analysis to explore potential biases in the comparisons with experiment. The collective findings indicate that considerations of force field compatibility through dielectric behavior is a potential strategy for future improvements in transfer processes between disparate environments.

**Electronic supplementary material:**

The online version of this article (doi:10.1007/s10822-016-9950-z) contains supplementary material, which is available to authorized users.

## Introduction

From the first SAMPL experiment in 2008 [1], through the most recent SAMPL experiments [2–5], classical explicit solvent alchemical transformation calculations have been regularly strong performers in blind predictions of hydration free energies. Such calculations usually involve static partial charges and atom parameters, but they often have angle and torsion flexibility and can properly sample relevant solute and solvent configurations, often important considerations in molecular transfer processes. The fixed nature of the parameters result in a notable dependence of the transfer free energy on the force field chosen to represent the solutes [6–12]. For the SAMPL5 challenge, the participants were given a more challenging task of predicting the distribution coefficient values ($\log D$) between water and cyclohexane of 53 different drug-like compounds [13]. By including non-aqueous condensed phases in the transfer process, the force field representation of the molecule needs to simultaneously correctly predict transfer into both phases from air in order to form a balanced thermodynamic cycle, otherwise there will be a systematic bias for one of the two (or more) phases.

We have recently been interested in exploring dielectric behavior as a possible route to improving force fields in classical molecular simulations [14, 15]. Modulation of standard force fields to correct for what is often flawed dielectric behavior could have beneficial consequences for molecule transferability in condensed-phase environments. The static dielectric constant has traditionally not been a core target experimental observable in force field development because of its more prohibitive computational cost, particularly for highly polar molecules where it tends to converge slowly [14]. Methods that have utilized the static dielectric constant in force field optimization have shown improved properties in comparisons with experiment [14, 16], in particular hydration free energies [15].

**Fig. 1:**
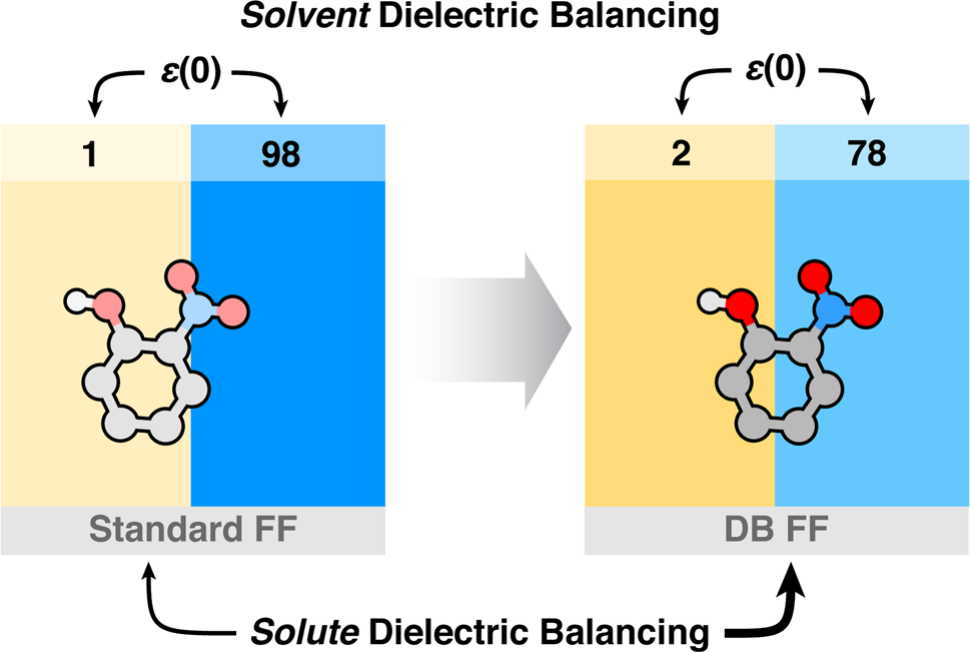
The dielectric balancing process involves a two part optimization of a transfer free energy estimation, *1* a solvent selection to ensure dielectric environments similar to those in experiments and *2* a solute force field adjustment to adapt the solute for the condensed phase. Commonly, in such transfer free energy calculations (*left side*), TIP3P and a classical cyclohexane have larger and smaller dielectric constants respectively than experimentally expected, and an under-polarized solute will partition into the nonpolar phase favorably by potential exclusion from the highly polar aqueous phase. In a dielectric balanced system (*right side*), systematic nonpolar biases should be reduced in favor of a more even accounting of solvation forces between the phases

With the potential importance of considering proper dielectric behavior for solutes and the solvents they reside within for accurate comparisons with and prediction of experimental properties, we have recently devised a strategy for explicit molecule transfer free energy calculations involving the balancing of component dielectric constants [17]. An illustration of this approach is presented in Fig. 1. We have observed general force fields to typically under-predict the static dielectric constant of neat liquids made using them, usually by around 50 % [14, 15]. Additionally, the solvent phases in typical explicit solvent transfer free energy calculations are TIP3P water ($\epsilon \left( 0\right) = 98$) and entirely nonpolar renditions of cyclohexane ($\epsilon \left( 0\right) =1$), phases with static dielectric constants notably different from the experimental values of 78.4 and 2.0 respectively [18]. Inserting a somewhat under-polarized solute in such exaggerated environments will likely lead to partitioning trends biased toward the nonpolar environment. Using more experimentally comparable solvents and appropriately polarized solutes could potentially remove such a hydrophobic bias and result in improved predictive accuracy.

We report here on an application of this force field dielectric balancing approach applied to the water-to-cyclohexane partitioning prediction challenge of the SAMPL5 experiment. We submitted two sets of predictions to the challenge, one where the solute and solvent environments were in proper balance and another where the solvent force fields are in dielectric balance with experiment but the solute is left unperturbed. We discuss the performance of these submissions, craft retrospective investigations to further clarify how these force field choices alter the expected outcomes for predicting experimental partitioning of drug-like molecules, and finish with a discussion on sources of error and future improvements.

## Computational methods

The water-to-cyclohexane distribution coefficients were prepared for the 53 solute molecules in the molecular transfer portion of the SAMPL5 event. As part of our dielectric balancing strategy (see Fig. 1), we calculated the air-to-solvent transfer free energies of all molecules in dielectrically corrected water and cyclohexane solvent environments and estimated the partition coefficient according to,

1 $$\log P = \frac{\Delta G_{wat}^\circ - \Delta G_{cyh}^\circ }{2.303RT}.$$

We do not perform corrections for tautomer, protonation, or aggregation states of the solute molecules in these calculations. Thus, we take these $\log P$ partition coefficient values as approximations of the experimental $\log D$ values in comparisons with experiment.

### Molecular models

The dielectrically corrected solvents were the fixed-charge H2O-DC water model [14], and for the nonpolar phase we used a united-atom cyclohexane with a small, fixed dipole, here referred to as CYH-DC. This model was optimized to reproduce the experimental static dielectric constant, density, and $\Delta H_{vap}$ following a previously published protocol [14]. Specific details about this optimization process, dipole placement decision, and resulting topology information are provided in the supplementary materials for this manuscript. In retrospective investigations, a limited set of additional calculations were performed using TIP3P water and a cyclohexane model created using GAFF parameters and AM1-BCC partial charges, referred to later as CYH [19–22]. Solute molecules were prepared by assigning GAFF parameters and AM1-BCC partial charges to the organizer provided PDB structures using the Antechamber package (Amber 14 version) [23]. Structures and topologies were converted to GROMACS format using ACPYPE python script [24], and each molecule was then solvated in the appropriate solvent in a rhombic-dodecahedral box with at least 1.2 nm of space between any solute atom and system box face.

In addition to using GAFF/AM1-BCC parameters, we modulated the solute non-bonded parameters following a recently tested internal protocol in order to balance the dielectric properties of the solute with the surrounding solvent [17].This modulation involves a 20 % magnification of the AM1-BCC partial charges and a corresponding linear inflation of the Lennard–Jones $\sigma$ parameters to maintain the proper liquid densities with the increased charge magnitudes. This degree of charge magnitude amplification has been seen as beneficial for neat liquid and molecular transfer properties by our group and others [15, 25], while the linear inflation is derived from automated dielectric optimization of small molecule functional groups. Here, the $\sigma _{\mathrm {LJ}}$ modulation amounts to a generally applied $\left( 5/12\right) \cdot \left| q_{\mathrm {new}}-q_{\mathrm {old}}\right|$ percent inflation for each atom of the molecule. We here refer to this modulated force field as dielectric balanced GAFF, or G-DB.

### Free energy calculations

The free energies associated with the molecular transfer of solutes from vacuum to either water or cyclohexane were computed using thermodynamic integration (TI). As is common practice [7, 26], the total solvation free energy in a given solvent ($\Delta G_{solv}$) was determined by the sum of 2 separated alchemical processes for calculating the nonpolar and polar components of solvation. An uncharged variant of the solute molecule was grown in the solvent to obtain the nonpolar contribution to solvation ($\Delta G_{solv,np}$), and the polar contribution ($\Delta G_{solv,pol}$) was determined by turning on the charges, less the intramolecular contributions to the electrostatic interactions. For the $\Delta G_{solv,np}$ TI calculations, $\lambda$ steps of (0.0 0.05 0.1 0.2 0.3 0.4 0.5 0.55 0.6 0.65 0.7 0.75 0.8 0.85 0.9 0.95 1.0) were used. For the $\Delta G_{solv,pol}$ TI calculations, 6 $\lambda$ steps evenly distributed from 0.0 to 1.0 where used. The simulations were performed using version 5.0.4 of the GROMACS package [27–31]. The temperature was held constant at 298.15 K with Langevin dynamics with an inverse friction coefficient of 2 ps, and a pressure of 1 atm was targeted using the Parrinello-Rahman barostat. Following 300 ps of equilibration, each TI window was sampled for 5 ns using a 2 fs timestep for integrating the equations of motion with the leap-frog algorithm. All bonds to hydrogen atoms were constrained using P-LINCS [32]. Lennard–Jones interaction were computed using a shifted cutoff at 1.2 nm, and energy and pressure long-range dispersion corrections were applied. Interactions between charges where computed using PME with 0.12 grid spacing and a real-space cut-off of 1.2 nm.

## Results and discussion

We contributed two submissions to the SAMPL5 event, submission numbers 36 and 42. Both of these used the dielectrically balanced solvents discussed in methods Sect. 2.1. While one (submission 42) used typical GAFF parameters with AM1-BCC partial charges, the other (submission 36) used dielectrically balanced GAFF/AM1-BCC, or G-DB, parameters. Going into the challenge, we expected the G-DB results to potentially be an improvement over the GAFF free energy perturbation calculations performed by one of the organizers. We also expected the imbalanced submission (#42) to perform more poorly than G-DB since the solute force field had not been adjusted to match its balanced condensed-phase environment.

### Compatible solvent and solute force fields are critical for prediction accuracy

**Fig. 2:**
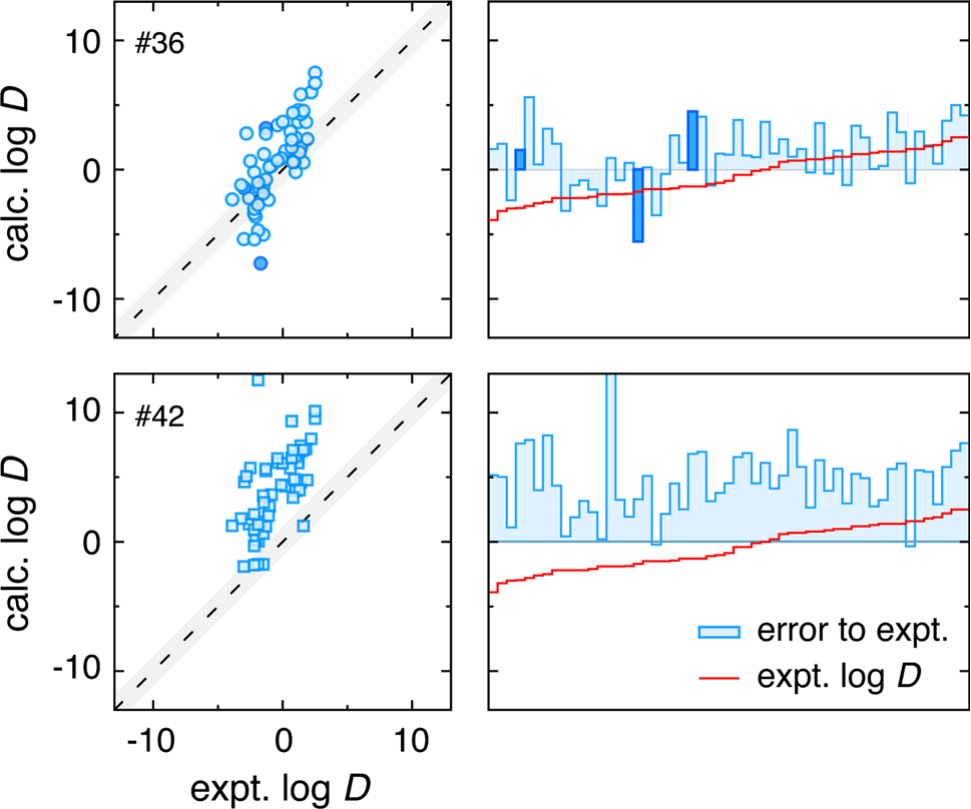
Correlation and error analysis of the two prospective SAMPL5 submissions from this effort. #36 is the preliminary dielectric balanced system, while #42 is the standard GAFF + AM1-BCC charges solute representation in the dielectric balanced solvents. As expected, submission #36 is more accurate than #42 because the solute FF and solvent environments are more compatible. Both submissions show a positive error slope on the right side plots indicating an exaggerated prediction behavior relative to experiment. The *darkened bars* and points in submission #36 results indicate the 3 solutes with topology errors that are corrected in later retrospective investigations

Figure 2 shows correlation scatter plots and error charts for submissions 36 and 42 in comparisons with the organizers’ supplied experimental values [13]. These results confirmed our expectations for the two submissions, though the errors observed for the G-DB calculations are greater than expected given the errors we have observed for calculations involving smaller molecules [17]. However, one might generally expect errors to grow with increasing numbers of functional groups and more varied local environments [33]. Regardless, the performance was quite respectable for G-DB, with consistent presence in the top 10–20 % of predictors across the provided error metrics for the full set of 53 molecules. Highlights include the 2nd ranked Pearson correlation coefficient (R = 0.75), 4th ranked Kendall’s tau coefficient ($\tau$ = 0.57), and 6th ranked RMS error (RMSE = 2.6) of the 62 submissions that reported results for all molecules. Care should be taken to not read to deeply into such performance metrics, as 53 molecules is not a particularly large evaluation set. Nevertheless, it is an encouraging result given the classical, fixed-charge nature of the models and lack of any effort placed in correcting for experimental measurement non-idealities, such as solvent mixing and pKa corrections.

The blue bars of error plots on the right side of Fig. 2 show the signed error relative to the experimental $\log D$ values, which are overlaid as a red line ordered by increasing $\log D$. Note that the calculations for the 3 bars and points labeled in darker blue had minor topology errors discovered after submission, though corrected results do not significantly alter the error metrics beyond a 0.2 positive shift in the mean signed error (MSE). Corrected results and error assessments are presented in all retrospective plots and discussions. The G-DB results show an interesting trend of errors shifting from generally negative to generally positive values as the experimental $\log D$ values increase. This is the result of a positive correlation slope, indicating that near the extremes of the reported experimental $\log D$ values the absolute predictions are greater in magnitude than experiment. Though less apparent in the error plots of the GAFF solute results in Fig. 2, this trend is also present.

Comparison of these G-DB and GAFF solute results in the dielectrically balanced solvent system demonstrates the importance of compatible solute and solvent force fields in molecular transfer calculation accuracy. While the G-DB results appear to be mostly balanced, there is a modest bias (MSE = 1.1 units of $\log D$) toward solvation in the cyclohexane phase. The GAFF solute results show a dramatically increased bias for the cyclohexane phase (MSE = 4.6 units of $\log D$), this because the solutes are severely under-polarized for a condensed phase environment with corrected dielectric constants. As CYH-DC is slightly polar, it becomes a “catch all” universal solvent for most of SAMPL5 solutes. The rest of the performance metrics are similarly poor for GAFF solutes in the dielectrically balanced environment because of this dramatic and systematic shift. If high predictive accuracy is the goal, balanced solute and solvent force fields are essential.

### Polarizing solutes for the condensed phase reduces systematic biases in partitioning

**Fig. 3:**
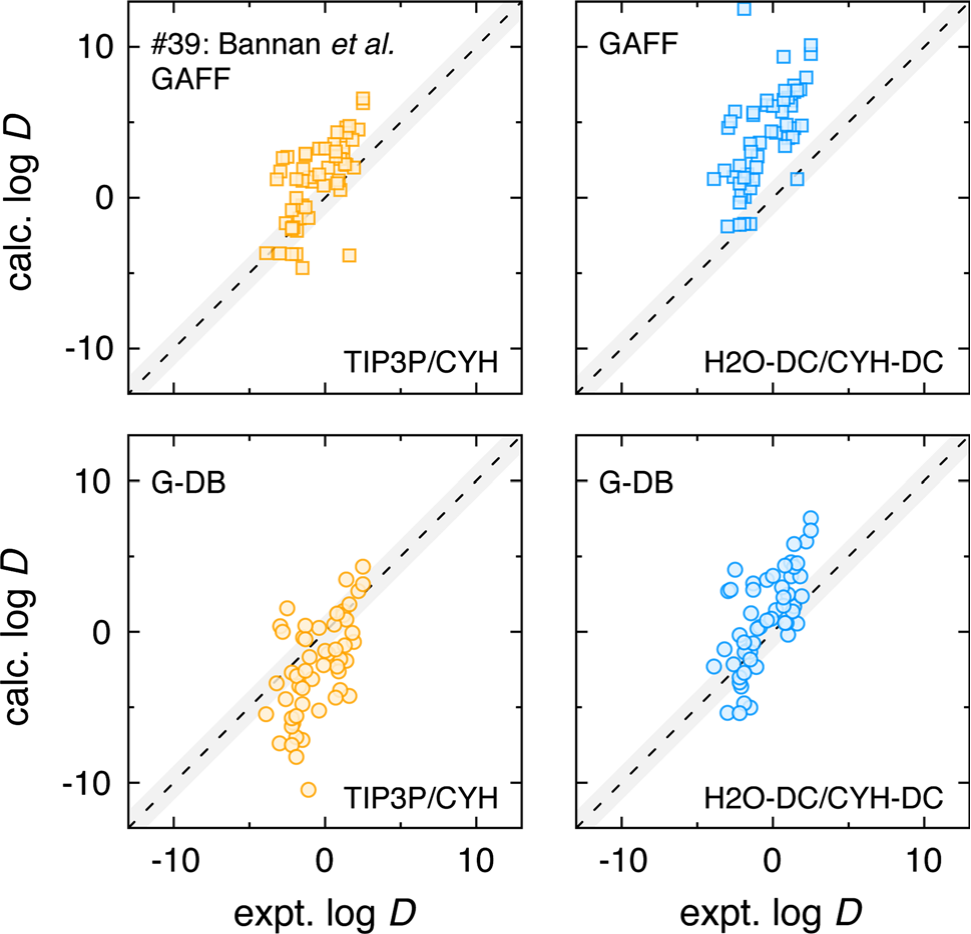
Correlation scatter plots for retrospective investigations of the GAFF (*top plots*) and G-DB (*bottom plots*) solutes in TIP3P/CYH (*orange points*) and H2O-DC/CYH-DC (*blue points*) environments. The submission #39 data was provided by one of the organizers, and the other 3 plots itemize the other solute/solvent combinations possible when considering changes in solvent and solute topologies. If the solute is under-polarized, as in the case of GAFF solutes in either solvent set, we see a systematic upward shift in the distributions, notably favoring the cyclohexane phase. The polarized G-DB solutes significantly favor TIP3P over the very nonpolar CYH, seen in the downward shift of the distribution. The dielectrically balanced system of G-DB solutes in H2O-DC/CYH-DC has a slightly upward systematic bias, though it is notably less than that seen in submission 39

To better investigate the importance of balancing the polarity of solutes with their solvent environments, we retrospectively considered the possible combinations of solute and solvent environments with respect to changes in their dielectric properties. One of these combinations was already performed by the organizers (submission #39) and graciously provided for this comparison. Figure 3 shows correlation plots of calculated values for GAFF and G-DB solutes in the TIP3P/CYH and H2O-DC/CYH-DC solvent environments with experimental values. The shape of the clustering of points is expectedly similar in all four plots, but the degree of vertical shift is what distinguishes these sets. When a solute/solvent combination favors the cyclohexane phase, we observe an upward vertical shift in the point cluster. When a solute/solvent combination favors the aqueous phase, we observe a downward vertical shift in the point cluster. Notably, the dielectrically balanced set improves on simply using GAFF solutes with the very nonpolar CYH by eliminating some of the systematic bias (MSE = 1.6 units of $\log D$) towards partition into CYH. This was the original goal of this dielectric balancing undertaking. The dielectrically balanced set still has a bias towards the cyclohexane phase (MSE = 1.3 units of $\log D$), though this improvement is only moderately significant given bootstrap sampling showing an uncertainty of 0.3 units of $\log D$. We believe that pKa correction considerations can reduce this bias further, as such corrections should help stabilize the solute partitioning into the aqueous phase.

The off-diagonal combinations further demonstrate the importance of a balanced approach to the polarity of solute molecules and their solvent environments. On seeing the results of GAFF in the typical solvent combination of TIP3P and a general CYH, one might be tempted to simply polarize the solute to appropriately “fix” the solute for the condensed phase environment. A version of this is shown in the plot of G-DB in TIP3P/CYH, and there is a significant shift towards the aqueous phase (MSE = -1.8). We believe that a more encompassing approach, one that considers the proper polarity of the environment as well as the solute, is more beneficial. This position is supported by the results seen here.

### Null hypothesis for $\log D$ values provides a challenging test

When analyzing predictive performance of a computational technique, it is often informative to consider a theoretical baseline expectation for accuracy given no specific knowledge of the makeup of a given sample. In molecular transfer processes, such a “null hypothesis” would not assume any knowledge of the structure or chemistry of the solutes and solvents involved in the experiment. For a computational technique to provide predictive value, it should be able to translate knowledge about the structure of the solutes and environments into predicted values and value trends that perform better than the baseline given no structural information. A well-defined null hypothesis for molecular transfer processes is that a given solute distributes equally between the two phases of interest, or $\log D = 0$ for all solutes. This null hypothesis assumes no specific knowledge of the concentrations of the solute in each phase by claiming the molecule will have no preference. This null hypothesis is also a reasonable estimation for experiments that could potentially have limited dynamic range. To arrive at a distribution coefficient, accurate assessments of the solute concentrations in each of the phases would need to be made experimentally, and $\log D$ value of zero would be a minimum expectation for a reliable experimental result. In other words, if the technique can measure a solute concentration in one solvent phase, it should be able to measure a similar solute concentration in the other phase.

**Fig. 4:**
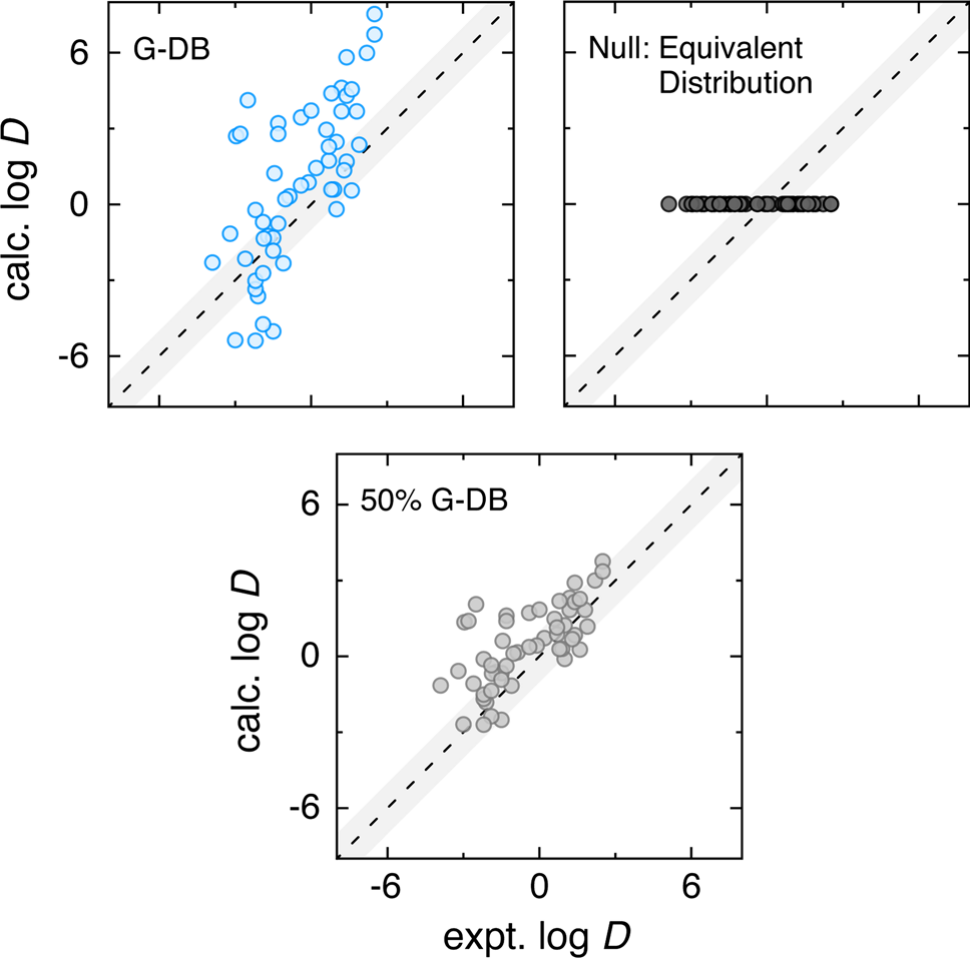
A comparison of our best performing SAMPL5 combination (*top left*), a null hypothesis (*top right*), and a variation that scales the magnitude of the solvation terms in both cyclohexane and water by 50 % (*bottom*). While the null hypothesis assumption has low error (AUE = 1.6 and RMSE = 1.8), it is not very predictive because of the flat trend. The 50 % scaling reduces the overly positive slope of our best predictor and decreases the errors by roughly 1 log unit (AUE = 1.2 and RMSE = 1.6) while maintaining a good predictive correlation (R = 0.75). The error values are notably better than the null test and about 0.5 log units better than the best submission in the SAMPL5 experiment

Figure 4 shows correlation plots for the proposed null hypothesis alongside our best performing prediction for the SAMPL5 experiment. As one should expect, assuming a constant $\log D$ of zero means that no trends in partition will be uncovered by the assumption. The unsigned and RMS error are however quite low: AUE = 1.6 and RMSE = 1.8. Surprisingly, these error values are even lower than the best performing submission, COSMO-RS by Klamt *et al.*, with AUE = 1.7 and RMSE = 2.1.

Why does this assumption pose such a difficult challenge, and what does this mean for the predictive value of the aggregate computational prediction efforts in SAMPL5? While this could simply be a chance outcome, the nature of the experimental data likely plays a role. The experimental values range from $\log D$ values of −4 to 3, a limited dynamic range of about 7 units of $\log D$ for these molecules, this compared to a dynamic range of nearly 14 for the G-DB predictions. The limited experimental range could be a consequence of organizer pre-selection of drug-like solutes that have reasonable favorability for both solvent phases, experimental pruning of the solute set to minimize error by avoiding the extremes of instrumental detection, and/or solvent impurities or mixing, i.e. water content in the cyclohexane phase and vice versa. The experimental values are also expected to be equilibrium results, and this would include variation in the conformation, aggregation, and protonation states of the structures.

In an attempt to address these potential considerations, we did preliminary screening of pKa corrections, like those provided by Schrödinger’s Epik tool [34], as well as select transfer calculations into mixed solvent states. Testing of preliminary pKa corrections used by other participants in and the organizers of the SAMPL5 challenge resulted in a systematic increase in the favorability for several molecules to partition into the aqueous phase. While this improved G-DB predictive correlation for molecules that experimentally favor the aqueous state by further stabilizing them, it unfortunately increased the positive slope of the $\log D$ trend and increased the dynamic range of the predictions, furthering the trend gap with the experimental data rather than closing the gap. The few attempted mixed solvent simulations tended to add prediction noise, likely due to the finite nature of the systems in the TI calculations.

Surprisingly, the most successful corrective consideration was also the most empirical. We assumed that there was an inherent bias in the experimental data set, be it by experimental selection, pre-screening, or system non-idealities and simply tried to “say less” by scaling back our free energy magnitudes in both solvents by half. We would expect this preliminary scaling to improve predictions of experimental measurements where there could be water content in the cyclohexane phase and *vice versa*, this because such a system non-ideality will mediate interactions between the solutes and the environment, reducing the measured dynamic range. The correlation plot for this 50 % G-DB approach is shown in the bottom of Fig. 4. The errors relative to experiment are significantly reduced: AUE = 1.2 and RMSE = 1.6, and it is still well-correlated with experiment. This is a significant improvement over the null hypothesis and all prospective submissions in the SAMPL5 experiment.

What is the practical consequence of *doing better* by *saying less*? It likely indicates that this technique, and others positively correlated with experiment, do add practical predictive value by considering the solute structure and molecular interactions with it and the surrounding environment. The magnitude of the solvation effects might be overly enhanced due to the assumption of working with near-ideal system setups, and improvements will likely come from more detailed consideration of system non-idealities. Presumably, one could also “win” prediction events with expected narrow ranges in experimental quantities by systematically reducing prediction magnitude for techniques believed to over-stabilize solvation extremes, either by approximating such non-idealities through a constant scaling term or minimizing systematic outlier effects.

## Conclusion

We presented here predictions for water-to-cyclohexane partitioning in the SAMPL5 experiment using alchemical transformation free energy calculations. In these, we apply an added force field consideration that the solutes and solvents be balanced according to their effective static dielectric constants. In the case where we considered a proper balance according to this property, we achieved an enhanced accuracy and a general improvement in predictive quality for the blind prediction challenge. To better illustrate this potential improvement, we performed a retrospective analysis on the SAMPL5 molecules across a systematic series of solute and solvent combinations. The results highlighted how considering force field compatibility based on dielectric behavior is critical if one hopes to achieve quantitative accuracy in predictions of molecular transfer.

In addition to computational predictions, we performed a null hypothesis comparison on these molecules to assess the potential value added from our computational prediction. This null hypothesis poses a rather difficult challenge for predictions, at least with this particular set of molecules and experimental measurements provided by the organizers. We observed the null hypothesis prediction to have a lower error than all predictors; however, it lacked any predictive correlation with the experimental measurements. With the knowledge that a null hypothesis prediction performed well for this set of molecules, we retrospectively proposed a somewhat empirical prediction that halves the magnitude of the free energy estimations in each of the solvent phases. We observed this “say less” method to significantly outperform all the SAMPL5 submissions, as well as the null hypothesis, in the common accuracy metrics while retaining the predictive correlation of the base computational model. While this does not mean the accuracy of a typical method will always increase by saying less, such a strategy appears to target systematic errors in comparisons between the simplified TI calculations we used here and this specific set of experimental data.

Predictive comparison experiments like SAMPL are important venues for testing and evaluating techniques and ideas. In SAMPL5, the experiment posed a new form of molecular transfer challenge, one that tests the limits of classical fixed-charge force fields. Our results were encouraging as they indicated further refinement by balancing of force fields through material dielectric constants could represent a somewhat straightforward path to improved quantitative accuracy in molecular transfer. It is also expected that further improvements will likely depend on detailed consideration of non-ideal system effects, like solvent mixing, solute dimerization, and solute protonation/deprotonation.

## Electronic supplementary material

Below is the link to the electronic supplementary material.

Supplementary material 1 (pdf 109 KB)

## References

[1] Nicholls A, Mobley DL, Guthrie JP, Chodera JD, Bayly CI, Cooper MD, Pande VS (2008). Predicting small-molecule solvation free energies: an informal blind test for computational chemistry. J Med Chem.

[2] Guthrie JP (2009). A blind challenge for computational solvation free energies: introduction and overview. J Phys Chem B.

[3] Geballe MT, Skillman AG, Nicholls A, Guthrie JP, Taylor PJ (2010). The SAMPL2 blind prediction challenge: introduction and overview. J Comput Aided Mol Des.

[4] Skillman AG (2012). SAMPL3: blinded prediction of host-guest binding affinities, hydration free energies, and trypsin inhibitors. J Comput Aided Mol Des.

[5] Mobley DL, Wymer KL, Lim NM, Guthrie PJ (2014). Blind prediction of solvation free energies from the SAMPL4 challenge. J Comput Aided Mol Des.

[6] MacCallum JL, Tieleman DP (2003). Calculation of the water-cyclohexane transfer free energies of neutral amino acid side-chain analogs using the opls all-atom force field. J Comput Chem.

[7] Shirts MR, Pitera JW, Swope WC, Pande VS (2003). Extremely precise free energy calculations of amino acid side chain analogs: comparison of common molecular mechanics force fields for proteins. J Chem Phys.

[8] Oostenbrink C, Villa A, Mark AE, van Gunsteren WF (2004). A biomolecular force field based on the free enthalpy of hydration and solvation: the gromos force-field parameter sets 53a5 and 53a6. J Comput Chem.

[9] Mobley DL, Dumont E, Chodera JD, Dill KA (2007). Comparison of charge models for fixed-charge force fields: small-molecule hydration free energies in explicit solvent. J Phys Chem B.

[10] Shivakumar D, Williams J, Wu Y, Damm W, Shelley J, Sherman W (2010). Prediction of absolute solvation free energies using molecular dynamics free energy perturbation and the opls force field. J Chem Theory Comput.

[11] Kehoe CW, Fennell CJ, Dill KA (2012). Testing the semi-explicit assembly solvation model in the SAMPL3 community blind test. J Comput Aided Mol Des.

[12] Mobley DL, Liu S, Cerutti DS, Swope WC, Rice JE (2012). Alchemical prediction of hydration free energies for SAMPL. J Comput Aided Mol Des.

[13] Rustenburg AS, Dancer J, Lin B, Ortwine DF, Mobley DL, Chodera JD (2016) Measuring experimental cyclohexane/water distribution coefficients for the sampl5 challenge, ibid 10.1007/s10822-016-9971-7 PMC5209288 27718028

[14] Fennell CJ, Li L, Dill KA (2012). Simple liquid models with corrected dielectric constants. J Phys Chem B.

[15] Fennell CJ, Wymer KL, Mobley DL (2014). A fixed-charge model for alcohol polarization in the condensed phase, and its role in small molecule hydration. J Phys Chem B.

[16] Wang L-P, Martinez TJ, Pande VS (2014). Building force fields: an automatic, systematic, and reproducible approach. J Phys Chem Lett.

[17] Paranahewage SS, Gierhart CS, Fennell CJ (2016) Dielectric balancing improves liquid-state properties and balances molecular transfer. Manuscript in preparation, Department of Chemistry, Oklahoma State University, Stillwater, USA

[18] Lide DR (2004). CRC Handbook of Chemistry and Physics.

[19] Jorgensen WL, Chandrasekhar J, Madura JD, Impey RW, Klein ML (1983). Comparison of simple potential functions for simulating liquid water. J Chem Phys.

[20] Wang J, Wolf RM, Caldwell JW, Kollman PA, Case DA (2004). Development and testing of a general amber force field. J Comput Chem.

[21] Jakalian A, Bush BL, Jack DB, Bayly CI (2000). Fast, efficient generation of high-quality atomic charges. AM1-BCC model: I. method. J Comput Chem.

[22] Jakalian A, Jack DB, Bayly CI (2002). Fast, efficient generation of high-quality atomic charges. AM1-BCC model: II. parameterization and validation. J Comput Chem.

[23.] Wang J, Wang W, Kollman PA, Case DA (2006). Automatic atom type and bond type perception in molecular mechanical calculations. J Mol Graphics Model.

[24] Sousa da Silva AW, Vranken WF (2012). ACPYPE—AnteChamber PYthon Parser interfacE. BMC Res Notes.

[25] Dodda LS, Vilseck JZ, Cutrona KJ, Jorgensen WL (2015). Evaluation of cm5 charges for nonaqueous condensed-phase modeling. J Chem Theory Comput.

[26] Mobley DL, Barber AE, Fennell CJ, Dill KA (2008). Charge asymmetries in hydration of polar solutes. J Phys Chem B.

[27] Berendsen HJC, van der Spoel D, van Drunen R (1995). GROMACS: a message-passing parallel molecular dynamics implementation. Comput Phys Commun.

[28] van der Spoel D, Lindahl E, Hess B, Groenhof G, Mark AE, Berendsen HJC (2005). Gromacs: fast, flexible, and free. J Comput Chem.

[29] Hess B, Kutzner C, van der Spoel D, Lindahl E (2008). Gromacs 4: algorithms for highly efficient, load-balanced, and scalable molecular simulation. J Chem Theory Comput.

[30] Pronk S (2013). Gromacs 4.5: a high-throughput and highly parallel open source molecular simulation toolkit. Bioinformatics.

[31] Abraham MJ, Murtola T, Schulz R, Páll S, Smith JC, Hess B, Lindahl E (2015). Gromacs: high performance molecular simulations through multi-level parallelism from laptops to supercomputers. SoftwareX.

[32] Hess B (2008). P-LINCS: a parallel linear constraint solver for molecular simulation. J Chem Theory Comput.

[33] Dill KA (1997). Additivity principles in biochemistry. J Biol Chem.

[34] Shelley JC, Cholleti A, Frye L, Greenwood JR, Timlin MR, Uchimaya M (2007). Epik: a software program for pka prediction and protonation state generation for drug-like molecules. J Comput Aided Mol Des.

