# Supplementary material for: Predicting water-to-cyclohexane partitioning of the SAMPL5 molecules using dielectric balancing of force fields
Source: J Comput Aided Mol Des. 2016 Aug 29;30(11):1059–65. doi: 10.1007/s10822-016-9950-z (PMC5206264; doi:10.1007/s10822-016-9950-z)
Supplement: Supplementary file 1 — Supplementary material 1 (pdf 109 KB) [file 10822_2016_9950_MOESM1_ESM.pdf]

Supplementary materials for: Predicting water-to-cyclohexane partitioning of the SAMPL5 molecules using dielectric balancing of force fields

S. Shanaka Paranahewage, Cassidy S. Gierhart, and Christopher J. Fennell

The following pages of this supporting information document contain topology information for the CYH-DC united atom cyclohexane model and tabulated results for the transfer free energy calculations presented in the main manuscript.

SI 1 CYH-DC model

An automated optimization procedure (see Fennell, C. J.; Li, L.; Dill, K. A. *J. Phys. Chem. B*, **116**(23), 6936, (2012)) was used for developing a simple cyclohexane model (CYH-DC) that directly reproduces the dielectric constant of the neat liquid. This is done by mimicking the induced polarization of the molecule in the condensed phase with a small permanent dipole moment. A united-atom form was chosen for computational efficiency in both the optimization process and in subsequent transfer calculations. To start the optimization, the bonded topology of the carbons in the six-membered cyclohexane ring was assembled using GAFF parameters. Two carbons at opposite sides of the chair-conformation ring were selected and given charges of ± 0.1, while the remaining four carbon atoms were given no charge. The mass of each carbon atom site was also increased to 14.026 amu, that of a united atom CH_2 group. A liquid state simulation of 200 of these initial cyclohexane molecules was created, and the automated dielectric correction tool was left to uniformly scale the charges, σ_{LJ}, and ϵ_{LJ} parameters to capture the experimental $\varepsilon(0)$, ρ, and ΔH_{vap} respectively. The resulting charge magnitude of the two opposing methylene centers was 0.08409, and all methylene sites were assigned $\sigma_{\mathrm{LJ}} = 3.9604$ Å and $\epsilon_{\mathrm{LJ}} = 0.1227$ kcal/mol.

SI 2 SAMPL5 results

The following tables present the submission results for the dielectric balanced (#36) and imbalanced (#42) contributions to the SAMPL5 solvation challenge, and the retrospective 50% G-DB $\log D$ results.

Table 1 Submission 36 cyclohexane and water ΔG_{pol}, ΔG_{np}, and ΔG_{solv} values, and the resulting $\log D$ predictions.

	CYH-DC Solvent						H2O-DC Solvent						
SAMPL ID	ΔG_{pol}	Error	ΔG_{np}	Error	ΔG_{solv}	Error	ΔG_{pol}	Error	ΔG_{np}	Error	ΔG_{solv}	Error	$\log D$
SAMPL5_002	-1.76	0.01	-17.03	0.04	-18.79	0.04	-18.25	0.03	1.76	0.06	-16.49	0.07	1.69
SAMPL5_003	-1.49	0.02	-14.53	0.03	-16.02	0.04	-14.10	0.02	1.29	0.05	-12.81	0.06	2.36
SAMPL5_004	-2.72	0.01	-18.45	0.04	-21.17	0.04	-15.10	0.03	2.11	0.07	-13.00	0.07	5.99
SAMPL5_005	-1.16	0.02	-19.13	0.04	-20.29	0.04	-20.02	0.14	0.17	0.09	-19.85	0.17	0.32
SAMPL5_006	-0.86	0.01	-13.29	0.04	-14.15	0.04	-15.44	0.07	1.58	0.06	-13.86	0.09	0.21
SAMPL5_007	-0.17	0.01	-18.50	0.04	-18.67	0.05	-11.84	0.04	1.11	0.07	-10.73	0.08	5.82
SAMPL5_010	-1.42	0.02	-15.70	0.04	-17.12	0.04	-27.80	0.04	0.79	0.07	-27.01	0.08	-7.25
SAMPL5_011	-1.37	0.02	-17.37	0.05	-18.74	0.05	-21.09	0.04	0.38	0.05	-20.70	0.07	-1.44
SAMPL5_013	-1.11	0.01	-23.43	0.04	-24.54	0.04	-26.80	0.04	0.47	0.07	-26.34	0.08	-1.32
SAMPL5_015	-1.23	0.03	-15.33	0.07	-16.56	0.08	-16.81	0.17	-0.04	0.05	-16.86	0.18	-0.22
SAMPL5_017	-0.74	0.01	-20.57	0.08	-21.31	0.08	-12.20	0.30	1.16	0.07	-11.04	0.31	7.53
SAMPL5_019	-0.52	0.02	-20.20	0.07	-20.72	0.07	-15.85	0.37	1.42	0.07	-14.43	0.38	4.61
SAMPL5_020	-0.99	0.04	-16.67	0.05	-17.67	0.06	-19.40	0.03	2.48	0.06	-16.92	0.07	0.55
SAMPL5_021	-0.70	0.02	-16.09	0.04	-16.79	0.04	-12.52	0.03	0.74	0.05	-11.78	0.06	3.68
SAMPL5_024	-5.24	0.01	-22.23	0.04	-27.47	0.05	-25.96	0.04	1.85	0.07	-24.11	0.08	2.47
SAMPL5_026	-1.83	0.01	-13.60	0.03	-15.42	0.03	-20.79	0.16	2.43	0.10	-18.36	0.19	-2.15
SAMPL5_027	-0.65	0.01	-15.04	0.03	-15.70	0.03	-17.09	0.04	-0.45	0.05	-17.54	0.06	-1.35
SAMPL5_033	-1.25	0.01	-19.91	0.05	-21.16	0.05	-19.07	0.06	2.93	0.09	-16.14	0.10	3.68
SAMPL5_037	-1.33	0.01	-14.74	0.03	-16.07	0.03	-21.72	0.03	-1.21	0.06	-22.93	0.07	-5.02
SAMPL5_042	-1.17	0.01	-17.93	0.04	-19.10	0.04	-24.16	0.05	1.89	0.06	-22.28	0.08	-2.33
SAMPL5_044	-2.43	0.01	-20.98	0.04	-23.41	0.04	-23.43	0.04	-0.24	0.06	-23.67	0.07	-0.19
SAMPL5_045	-1.40	0.01	-13.08	0.03	-14.48	0.03	-20.48	0.04	1.04	0.05	-19.43	0.06	-3.63
SAMPL5_046	-1.44	0.01	-18.86	0.04	-20.29	0.04	-19.66	0.06	1.33	0.06	-18.33	0.08	1.44
SAMPL5_047	-0.91	0.02	-18.11	0.04	-19.02	0.04	-14.73	0.02	0.42	0.06	-14.32	0.06	3.44
SAMPL5_048	-4.79	0.07	-20.69	0.07	-25.47	0.10	-25.01	0.08	0.34	0.07	-24.66	0.10	0.59
SAMPL5_049	-0.88	0.01	-14.21	0.04	-15.09	0.04	-14.24	0.03	1.00	0.05	-13.24	0.06	1.35
SAMPL5_050	-0.82	0.01	-15.45	0.04	-16.28	0.04	-17.69	0.39	-0.17	0.05	-17.86	0.39	-1.16
SAMPL5_055	-0.96	0.03	-12.00	0.03	-12.96	0.04	-15.63	0.03	0.17	0.05	-15.47	0.05	-1.83
SAMPL5_056	-1.69	0.02	-16.17	0.03	-17.86	0.04	-17.27	0.03	0.30	0.10	-16.97	0.11	0.66
SAMPL5_058	-1.33	0.01	-14.06	0.03	-15.39	0.04	-16.18	0.03	1.59	0.06	-14.59	0.07	0.59
SAMPL5_059	-0.91	0.01	-11.47	0.05	-12.38	0.05	-13.82	0.03	0.39	0.05	-13.42	0.06	-0.76
SAMPL5_060	-2.15	0.02	-14.33	0.05	-16.48	0.05	-19.64	0.03	0.02	0.05	-19.62	0.05	-2.30
SAMPL5_061	-0.46	0.02	-13.71	0.03	-14.18	0.04	-13.23	0.15	0.73	0.06	-12.50	0.16	1.23
SAMPL5_063	-1.29	0.01	-15.55	0.04	-16.84	0.04	-24.47	0.04	0.31	0.07	-24.17	0.08	-5.37
SAMPL5_065	-1.09	0.06	-38.27	0.07	-39.36	0.09	-39.91	0.10	2.91	0.11	-37.00	0.15	1.73
SAMPL5_067	-0.57	0.04	-15.59	0.06	-16.16	0.07	-15.00	0.20	3.22	0.09	-11.78	0.22	3.21
SAMPL5_068	-0.43	0.04	-19.40	0.04	-19.83	0.06	-16.29	0.03	2.33	0.11	-13.96	0.11	4.30
SAMPL5_069	-1.01	0.01	-21.16	0.04	-22.18	0.05	-19.52	0.05	1.16	0.07	-18.36	0.09	2.79
SAMPL5_070	-0.71	0.02	-16.36	0.05	-17.07	0.05	-14.90	0.04	4.04	0.07	-10.87	0.08	4.55
SAMPL5_071	-0.99	0.01	-17.01	0.04	-18.00	0.04	-19.31	0.05	2.48	0.06	-16.82	0.08	0.87
SAMPL5_072	-0.71	0.01	-15.18	0.05	-15.90	0.05	-15.94	0.07	4.07	0.08	-11.87	0.10	2.95
SAMPL5_074	-0.94	0.01	-19.65	0.04	-20.59	0.04	-25.08	0.27	-1.97	0.08	-27.05	0.28	-4.74
SAMPL5_075	-1.49	0.06	-16.83	0.08	-18.32	0.10	-18.15	0.19	3.64	0.07	-14.51	0.20	2.79
SAMPL5_080	-1.19	0.02	-15.30	0.03	-16.49	0.04	-22.54	0.03	-1.31	0.05	-23.85	0.06	-5.39
SAMPL5_081	-2.59	0.07	-16.93	0.05	-19.52	0.08	-26.97	0.23	2.88	0.07	-24.09	0.24	-3.35
SAMPL5_082	-0.34	0.08	-20.30	0.05	-20.64	0.10	-17.59	0.11	6.11	0.08	-11.47	0.14	6.72
SAMPL5_083	5.23	0.44	-43.53	0.10	-38.29	0.45	-44.02	1.42	4.77	0.29	-39.25	1.45	-0.70
SAMPL5_084	-1.11	0.09	-20.69	0.79	-21.80	0.79	-20.06	0.26	3.32	0.08	-16.74	0.27	3.71
SAMPL5_085	-1.23	0.01	-15.92	0.04	-17.14	0.04	-22.42	0.03	1.15	0.08	-21.27	0.08	-3.02
SAMPL5_086	-0.59	0.03	-22.49	0.06	-23.08	0.07	-23.53	0.38	3.55	0.08	-19.97	0.39	2.28
SAMPL5_088	-1.48	0.05	-16.78	0.05	-18.25	0.07	-25.09	0.08	3.12	0.07	-21.97	0.11	-2.72
SAMPL5_090	-0.19	0.02	-19.85	0.05	-20.05	0.05	-16.33	0.12	2.28	0.10	-14.06	0.16	4.39
SAMPL5_092	-0.82	0.30	-30.93	0.31	-31.75	0.43	-32.69	0.15	1.96	0.31	-30.72	0.34	0.75

Table 2 Submission 42 cyclohexane and water ΔG_{pol}, ΔG_{np}, and ΔG_{solv} values, and the resulting $\log D$ predictions.

	CYH-DC Solvent						H2O-DC Solvent						
SAMPL ID	ΔG_{pol}	Error	ΔG_{np}	Error	ΔG_{solv}	Error	ΔG_{pol}	Error	ΔG_{np}	Error	ΔG_{solv}	Error	$\log D$
SAMPL5_002	-1.31	0.01	-16.13	0.05	-17.43	0.05	-13.30	0.03	2.15	0.06	-11.15	0.07	4.61
SAMPL5_003	-1.05	0.01	-13.82	0.03	-14.86	0.03	-10.02	0.02	1.67	0.06	-8.35	0.06	4.78
SAMPL5_004	-2.00	0.01	-17.58	0.08	-19.58	0.08	-11.41	0.03	2.70	0.10	-8.71	0.11	7.97
SAMPL5_005	-1.00	0.02	-18.15	0.09	-19.14	0.09	-14.95	0.14	0.78	0.06	-14.17	0.15	3.65
SAMPL5_006	-0.65	0.01	-12.92	0.04	-13.57	0.04	-11.62	0.04	1.79	0.06	-9.83	0.08	2.74
SAMPL5_007	-0.16	0.01	-17.48	0.05	-17.63	0.05	-9.89	0.12	1.82	0.06	-8.07	0.14	7.01
SAMPL5_010	-0.80	0.01	-14.49	0.04	-15.29	0.04	-15.00	0.03	1.36	0.05	-13.64	0.06	1.21
SAMPL5_011	-0.79	0.01	-15.66	0.03	-16.46	0.04	-12.21	0.06	1.58	0.05	-10.63	0.08	4.27
SAMPL5_013	-0.80	0.01	-22.21	0.05	-23.01	0.05	-19.25	0.03	1.11	0.07	-18.13	0.07	3.58
SAMPL5_015	-1.32	0.03	-14.58	0.03	-15.90	0.04	-13.47	0.03	0.46	0.07	-13.00	0.07	2.12
SAMPL5_017	-0.72	0.01	-19.43	0.04	-20.15	0.04	-9.21	0.14	2.06	0.14	-7.15	0.20	9.53
SAMPL5_019	-0.39	0.01	-18.95	0.04	-19.33	0.04	-12.71	0.04	2.17	0.07	-10.55	0.08	6.44
SAMPL5_020	-0.75	0.01	-15.97	0.04	-16.72	0.04	-17.79	2.31	2.76	0.06	-15.04	2.31	1.23
SAMPL5_021	-0.49	0.01	-15.45	0.04	-15.94	0.04	-8.82	0.03	1.20	0.05	-7.62	0.06	6.10
SAMPL5_024	-3.70	0.01	-21.38	0.05	-25.08	0.05	-18.48	0.04	2.44	0.09	-16.03	0.10	6.63
SAMPL5_026	-1.29	0.01	-13.23	0.03	-14.52	0.04	-15.35	0.08	2.71	0.05	-12.64	0.10	1.37
SAMPL5_027	-0.53	0.01	-13.92	0.03	-14.45	0.03	-14.65	0.03	0.28	0.05	-14.38	0.06	0.05
SAMPL5_033	-0.95	0.01	-19.26	0.04	-20.21	0.04	-13.65	0.05	3.25	0.07	-10.40	0.08	7.19
SAMPL5_037	-0.98	0.01	-12.69	0.03	-13.67	0.03	-16.36	0.03	0.32	0.05	-16.04	0.06	-1.74
SAMPL5_042	-0.90	0.01	-17.34	0.04	-18.24	0.04	-17.61	0.04	2.14	0.07	-15.48	0.08	2.02
SAMPL5_044	-1.78	0.01	-20.01	0.10	-21.79	0.10	-16.68	0.03	0.27	0.06	-16.41	0.07	3.95
SAMPL5_045	-1.04	0.02	-12.50	0.03	-13.54	0.04	-14.71	0.03	1.41	0.04	-13.30	0.06	0.17
SAMPL5_046	-1.12	0.01	-17.92	0.05	-19.03	0.05	-15.23	0.06	2.06	0.06	-13.17	0.08	4.30
SAMPL5_047	-0.66	0.01	-17.49	0.04	-18.14	0.04	-10.44	0.02	0.67	0.10	-9.77	0.10	6.14
SAMPL5_048	-3.30	0.06	-19.93	0.04	-23.23	0.07	-17.51	0.11	0.87	0.07	-16.65	0.13	4.83
SAMPL5_049	-0.64	0.01	-13.61	0.03	-14.25	0.04	-10.10	0.03	1.32	0.05	-8.78	0.06	4.01
SAMPL5_050	-0.61	0.01	-14.71	0.03	-15.32	0.03	-13.29	0.12	0.42	0.06	-12.87	0.13	1.80
SAMPL5_055	-0.74	0.01	-11.32	0.03	-12.05	0.03	-11.56	0.02	0.36	0.05	-11.20	0.05	0.63
SAMPL5_056	-0.83	0.01	-15.07	0.04	-15.91	0.04	-9.32	0.03	0.90	0.05	-8.42	0.06	5.49
SAMPL5_058	-0.94	0.01	-13.59	0.04	-14.53	0.04	-11.51	0.02	1.66	0.05	-9.85	0.06	3.43
SAMPL5_059	-0.74	0.01	-10.81	0.04	-11.55	0.04	-10.64	0.02	0.71	0.04	-9.93	0.05	1.19
SAMPL5_060	-1.52	0.03	-13.76	0.05	-15.28	0.06	-13.80	0.02	0.21	0.06	-13.59	0.07	1.24
SAMPL5_061	-0.34	0.01	-12.77	0.03	-13.11	0.03	-10.37	0.39	1.42	0.05	-8.96	0.40	3.04
SAMPL5_063	-0.95	0.01	-14.70	0.04	-15.65	0.04	-19.09	0.06	0.83	0.06	-18.26	0.09	-1.91
SAMPL5_065	-0.88	0.05	-36.64	0.06	-37.52	0.07	-28.61	0.11	3.84	0.09	-24.77	0.14	9.34
SAMPL5_067	-0.50	0.02	-15.10	0.11	-15.60	0.11	-11.66	0.18	3.54	0.07	-8.12	0.19	5.48
SAMPL5_068	-0.33	0.03	-18.58	0.04	-18.91	0.05	-11.48	0.09	2.71	0.16	-8.76	0.18	7.44
SAMPL5_069	-0.76	0.02	-20.60	0.06	-21.35	0.06	-15.29	0.12	1.64	0.10	-13.65	0.16	5.64
SAMPL5_070	-0.52	0.01	-15.92	0.05	-16.44	0.05	-11.00	0.03	4.27	0.07	-6.73	0.07	7.11
SAMPL5_071	-0.73	0.01	-16.27	0.06	-17.00	0.06	-13.91	0.03	2.91	0.16	-11.00	0.16	4.40
SAMPL5_072	-0.53	0.01	-14.50	0.05	-15.03	0.05	-11.66	0.05	4.41	0.06	-7.25	0.08	5.70
SAMPL5_074	-0.67	0.01	-18.46	0.05	-19.13	0.05	-19.96	0.10	-1.52	0.05	-21.49	0.11	-1.73
SAMPL5_075	-0.87	0.05	-16.03	0.06	-16.90	0.08	-14.28	0.14	4.29	0.11	-9.99	0.18	5.06
SAMPL5_080	-0.84	0.01	-13.91	0.63	-14.75	0.63	-16.69	0.03	-0.51	0.05	-17.21	0.05	-1.80
SAMPL5_081	-1.61	0.04	-15.97	0.06	-17.58	0.07	-21.11	0.24	3.09	0.06	-18.02	0.24	-0.32
SAMPL5_082	-0.32	0.06	-19.64	0.08	-19.96	0.10	-12.70	0.09	6.54	0.08	-6.16	0.12	10.11
SAMPL5_083	3.15	1.06	-41.53	0.10	-38.39	1.07	-27.48	1.32	6.17	0.17	-21.31	1.33	12.52
SAMPL5_084	-0.76	0.07	-19.92	0.12	-20.69	0.14	-16.11	0.23	3.71	0.10	-12.40	0.25	6.08
SAMPL5_085	-0.90	0.01	-15.10	0.05	-16.00	0.05	-16.29	0.03	1.58	0.05	-14.72	0.06	0.94
SAMPL5_086	-0.39	0.02	-21.05	0.06	-21.44	0.07	-17.12	0.24	4.51	0.17	-12.61	0.30	6.48
SAMPL5_088	-1.06	0.06	-15.85	0.07	-16.91	0.10	-18.68	0.10	3.59	0.06	-15.09	0.12	1.33
SAMPL5_090	-0.21	0.02	-18.88	0.05	-19.09	0.05	-12.31	0.09	2.89	0.07	-9.42	0.11	7.09
SAMPL5_092	-0.56	0.16	-29.52	0.18	-30.08	0.24	-24.28	0.12	2.99	0.14	-21.29	0.19	6.44

Table 3 Retrospective 50% G-DB log D calculations.

SAMPL ID	log D
SAMPL5_002	0.84
SAMPL5_003	1.18
SAMPL5_004	3.00
SAMPL5_005	0.16
SAMPL5_006	0.11
SAMPL5_007	2.91
SAMPL5_010	-0.62
SAMPL5_011	1.35
SAMPL5_013	-0.66
SAMPL5_015	-0.11
SAMPL5_017	3.76
SAMPL5_019	2.31
SAMPL5_020	0.27
SAMPL5_021	1.84
SAMPL5_024	1.23
SAMPL5_026	-1.08
SAMPL5_027	-0.68
SAMPL5_033	1.84
SAMPL5_037	-2.51
SAMPL5_042	-1.16
SAMPL5_044	-0.10
SAMPL5_045	-1.82
SAMPL5_046	0.72
SAMPL5_047	1.72
SAMPL5_048	0.30
SAMPL5_049	0.68
SAMPL5_050	-0.58
SAMPL5_055	-0.92
SAMPL5_056	2.06
SAMPL5_058	0.29
SAMPL5_059	-0.38
SAMPL5_060	-1.15
SAMPL5_061	0.61
SAMPL5_063	-2.69
SAMPL5_065	0.87
SAMPL5_067	1.61
SAMPL5_068	2.15
SAMPL5_069	1.40
SAMPL5_070	2.27
SAMPL5_071	0.43
SAMPL5_072	1.48
SAMPL5_074	-2.37
SAMPL5_075	1.40
SAMPL5_080	-2.70
SAMPL5_081	-1.68
SAMPL5_082	3.36
SAMPL5_083	-0.35
SAMPL5_084	1.85
SAMPL5_085	-1.51
SAMPL5_086	1.14
SAMPL5_088	-1.36
SAMPL5_090	2.19
SAMPL5_092	0.37
